# Encoded in Titanium: The Digital DNA of Forensic Dentistry

**DOI:** 10.7759/cureus.83669

**Published:** 2025-05-07

**Authors:** Sourabh Khandelwal, Rajeev Srivastava, Ranjan Mani Tripathi, Mahendra Dave, Ankush Jain, Sarthak Shrivastav

**Affiliations:** 1 Department of Prosthodontics and Crown and Bridge, Index Institute of Dental Sciences, Malwanchal University, Indore, IND; 2 Department of Public Health Dentistry, Index Institute of Dental Sciences, Malwanchal University, Indore, IND

**Keywords:** dental implants, forensic dentistry, forensic odontology, human identification, implant recognition software, postmortem identification

## Abstract

Forensic odontology greatly aids the identification of unknown people, particularly in situations where more conventional techniques like fingerprinting and iris recognition are impractical. This paper examines the increasing significance of dental implants in forensic research, emphasizing their special benefits, such as their resilience to high temperatures, longevity, and capacity to preserve identifying characteristics like batch or serial numbers. The article describes several identification approaches, including DNA profiling, radiographic imaging, lip and palatal print examination, and bite mark analysis, focusing on how dental implant analysis is incorporated into these techniques. The use of Implant Recognition Software, radiographic comparison, and identification by the physical characteristics of implants are all considered crucial instruments for improving identification accuracy. Actual forensic cases and mass disaster situations demonstrate the usefulness of implant-based identification. Current issues, including the requirement for controlled implant databases and the absence of uniform documentation, are also covered in the evaluation. To maximize the forensic value of dental implants for identifying individuals, the process concludes by advocating for greater interdisciplinary collaboration and technological advancements.

## Introduction and background

The field of forensic odontology has continuously evolved and improved since the time of the Roman Empire. Because of his vital role in identifying the victims of the Paris fire, many people view Dr. Oscar Amoedo as the founder of forensic odontology [1]. A branch of dentistry called forensic odontology is dedicated to obtaining, analyzing, and presenting oral evidence in court. In situations such as violent assaults or major disasters, accurate identification becomes essential. When other means of identification are lacking, dental characteristics like restorations, missing teeth, and prosthetic appliances are crucial indicators of a person's identity [2]. This literature review underscores the crucial role of dentists in forensic odontology. Dental identification relies on various techniques, including bite mark analysis, prosthetic identification, cheiloscopy (lip print analysis), palatoscopy (palatal structure analysis), and dental implant research. Essential to this process are antemortem dental records, encompassing a comprehensive range of information such as dental and medical histories, written documentation, dental charts and diagrams, X-rays, laboratory findings, clinical photographs, referral notes, study models, prescriptions, and other relevant data [3].

Toolson and Taylor distinguished three primary fields of forensic odontology: (i) criminal, (ii) civil, and (iii) research [3]. The civil aspect addresses identification following catastrophic events like earthquakes, plane crashes, and train accidents, where remains are often severely compromised. This also covers negligence cases, insurance fraud, and age estimation, lacking official documentation like birth certificates. The identification of individuals, both perpetrators and victims, in homicide, suicide, and rape cases within the criminal justice system hinges on dental evidence, which encompasses techniques like bite mark analysis, cheiloscopy (the examination of lip prints), and palatal rugoscopy (the study of palatal ridges). In both civil and criminal trials, dentists play a critical role. To help with precise identification, they compare dental features, including bite marks, lip prints, and radiographic pictures, with antemortem documents like photos, cast models, and dental X-rays [4].

A critical area of dentistry that applies its fundamental ideas and expertise to criminal investigations and urban court cases is forensic dentistry, sometimes called forensic odontology. Its primary goals are to offer scientific analysis, support the investigation and prosecution of criminal activity, tackle pervasive social problems, and provide significant perspectives to anthropological and archaeological studies [5]. Unidentified remains are frequently found in a variety of situations, including natural death, homicide, suicide, drowning, and burning. Despite the potential for obvious clues, dental records and evidence are frequently crucial for precise identification [5].

Dental identification, primarily based on comparing antemortem and postmortem dental records, continues to be a highly reliable, well-established, and extensively utilized in forensic human identification. Rugoscopy (the study of palatal rugae patterns), cheiloscopy (the study of lip prints), tooth print analysis, radiographic imaging, photographic comparisons, and sophisticated molecular techniques like polymerase chain reaction (PCR) for the analysis of DNA from dental pulp are some of the methods used in forensic odontology [6]. In criminal investigations, mass casualty incidents, and cases involving extensively decomposed or damaged bodies, this technique is nevertheless essential, especially in situations when visual recognition is impractical or inappropriate. Dental identification is valued for its economy, speed, and precision.

A dental implant is a structure that is surgically inserted into the jawbone to support a dental prosthesis through a process known as osseointegration. In recent years, dental implants have grown in significance in forensic identification, particularly when the individual's entire dentition is composed of implants. Generally, dental implants are categorized based on their material properties, design, and attachment techniques [5,7]. Modern implants bond with the underlying bone tissue by a biological process known as osseointegration, which promotes union without causing a foreign body reaction. Materials such as ceramics and titanium are commonly used due to their biocompatibility and ability to facilitate bone incorporation. Advances in forensic technology, such as Implant Recognition Software, radiographic evaluation of implant features, and batch or serial number tracking, have made it much easier for forensic odontologists to identify individuals by comparing these results with antemortem dental data [7].

Problem statement

When there is significant decomposition or cremation, traditional forensic identification techniques frequently don't work. Because of their endurance and distinctive characteristics, dental implants present a significant opportunity for identification. However, their application in forensic practice is still restricted because of a lack of awareness, identifying tools, and standardization. This article examines dental implants' function as a trustworthy forensic identification tool.

Objectives

This article's primary goal is to highlight the value of dental implants as a dependable and long-lasting tool for forensic identification, especially when more conventional techniques are inadequate because of decomposition or combustion. Its goal is to investigate implants' radiographic and morphological traits useful for postmortem identification. Furthermore, the article aims to assess how well radiography methods and implant recognition algorithms can identify implant systems. It also emphasizes the importance of uniform paperwork and implant tracking to improve forensic investigations. To increase the precision and effectiveness of human identification, the study suggests incorporating implant-based identification into accepted forensic procedures.

## Review

Materials and methods

**Figure 1:**
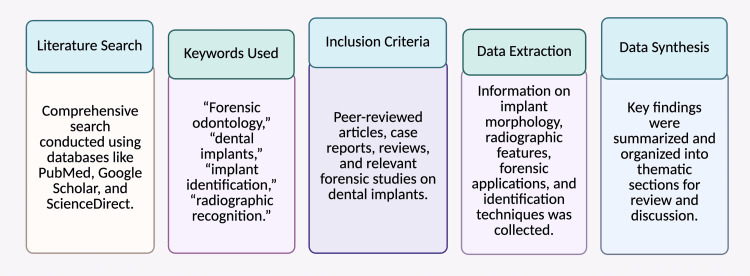
Flow chart illustrating the methodology of the study Note: This figure has been drawn using the premium version of BioRender (https://BioRender.com/3ilcw8d), agreement license number LO287O9HZ4, accessed April 24th, 2025 [6]. Illustration Credit: Dr. Vishnu Desai. Permission granted for the use.

The foundation of this review paper is a thorough examination of the body of research on dental implants' function in forensic identification. A comprehensive search was carried out using terms including "forensic odontology," "dental implants," "implant identification," and "radiographic recognition in forensics" across several research databases, including PubMed, Google Scholar, and ScienceDirect. Peer-reviewed publications, case studies, review articles, and reference books were incorporated to collect pertinent data. The selection of articles was based on their significance for forensic uses of dental implants, including morphological features, radiographic identification, incineration resistance, and traceability via batch or serial numbers. The data were combined to emphasize existing procedures, technological developments, constraints, and suggestions for the future in implant-based forensic identification (Figure 1).

Forensic odontology's role in human identification

A relatively new subject that uses dental knowledge to support the legal system is forensic odontology. It emphasizes three main areas: (1) evaluations for diagnosis and treatment, (2) recognizing people, and (3) looking at, evaluating, and analyzing bite marks [8]. The process of human identification involves matching the physical attributes of an unidentified body (postmortem data) with the distinguishing features of a missing individual (ante-mortem data) [9]. Visually identifying deceased people is usually done by a relative or acquaintance who knew the person during their life. Typically, this process entails identifying physical traits, facial features, or personal belongings. However, when the body has experienced substantial modifications as a result of burning, decomposition, or other post- and peri-mortem changes, this approach becomes unreliable and inappropriate. Visual identification is prone to inaccuracy in these situations. DNA profiling, footprint and fingerprint analysis, and the investigation of dental and medical features, such as scars, tattoos, birthmarks, implants, amputations, and prosthetic devices, are scientifically validated means of human identification [9].

Ante-mortem data must be available for dental identification to be successful. Comprehensive dental exams, precise documentation, and the safe preservation of results pertaining to the hard and soft oral tissues, radiographs, study models, clinical photos, and other pertinent documents are all crucial to this procedure. When such information is available, it facilitates precise identification by comparing the person's dental traits during life with those seen after death [10]. Forensic dentistry can help identify a person in cases where dental records are unavailable by creating a profile of the person's personality throughout their lifespan. Unique eating habits, dietary patterns, socioeconomic level, and an estimate of the person's age at death may all be included in this profile [11]. The tooth development and eruption timeline is the primary basis for dental age assessment, and it works particularly well for those up to about fifteen years old. After this age, dental aging depends on lifelong developments, including tooth wear (attrition), cementum production, and the growing transparency of the tooth roots [12].

The dentist's role in forensic identification

A simple matching technique is to use routine identification tasks. For local native authorities, mass fatality incidents pose a significant issue. An ante-mortem, postmortem, and reconciliation team must be part of a hierarchical structure to identify deceased victims in those situations. Many mass tragedies have been resolved with the help of forensic odontologists. Perhaps the most notable instance of forensic orthodontists' ability to quickly and accurately identify various victims is the 2004 Indian Ocean tsunami wave. The identification of tsunami victims in Thailand was aided by the fact that about ½ of the victims were identified using dental traits alone [13].

Bitemark analysis in forensic odontology

Teeth injuries imprinted on surfaces such as skin frequently have a distinct and identifiable pattern. Bite marks are patterned injuries that are useful to legal authorities because they can help recreate past events by offering insights about the biting incident's circumstances [14]. Bite marks, for example, may indicate a violent interaction between the victim and the offender, which may disclose the offender's intention, be it sexual assault, child abuse, or other types of violence. Furthermore, bite marks are distinctively patterned injuries that, to varying degrees of accuracy, can be used to identify the perpetrator. Investigators can determine whether there may be a match by examining the bite marks' location and size and contrasting them with the dental anatomy of a suspect [15]. Recent investigations have employed 3D automated comparisons of bite marks and teeth. This advanced technique addresses perspective distortion, a significant challenge in bite mark identification, by flattening 3D objects as 2D images [16]. The Salem Witch Trials in 1692 are the oldest known case of bite mark identification. Theodore (Ted) Bundy, a serial murderer, was found guilty in part because of bite mark analysis given in a U.S. court in a landmark case that brought bite mark evidence into the spotlight in the courtroom [17]. In a case where forensic odontology was crucial, a death sentence was given for the first time in India's criminal prosecution history. The forensic odontologist in the Delhi gang rape case was able to connect the victim's bite marks to the tooth patterns of two of the defendants [18].

Lip prints (cheiloscopy) in forensic identification

Cheiloscopy is a forensic identification technique that employs lip print analysis to determine an individual's identity [19]. When utilizing them for antemortem comparison, tooth loss or decaying dental restorations might make it challenging to match dental records accurately with postmortem records. Like fingerprints, palm prints, and footprints, each person's lip print is distinct and doesn't change throughout their lifetime. Because lip prints include unique grooves and furrows, they are useful in forensic investigations. These prints can be obtained directly from the deceased's lips or indirectly from items found at a crime scene, like windows, doors, clothing, cups, glasses, and cigarettes. A lip print's design might change depending on whether the mouth is open or closed. In an open-mouth position, the grooves are typically less distinct and more difficult to read, whereas in a closed-mouth position, they appear well-defined and easier to analyze [20]. This characteristic aids in determining a person's gender [21].

Rugoscopy/palatoscopy in forensic identification

To determine a person's identity, palatoscopy, sometimes called palatal rugoscopy, is a forensic identification technique that examines the distinctive ridges on the roof of the mouth called palatal rugae. Edmond Locard originally suggested using lip prints in forensic science in France in 1932. The calcified mesenchymal tissue around the palate is the source of palatal rugae, which form during the third month of intrauterine development [18]. Between weeks 12 and 14 of pregnancy, the exact positioning, form, and direction of the palatal rugae are usually determined. These structures last until the oral mucosal tissues start to degrade after death and are extraordinarily durable throughout an individual's lifetime. Palatal rugae are a useful identifying tool because of their distinctive and durable qualities, especially when fingerprints or tooth hard tissue records are degraded or unavailable [22]. Although palatal rugae are usually constant, age and other environmental or clinical conditions might cause alterations in their patterns. These include periodontal procedures, cleft palate surgeries, orthodontic treatments, tooth extractions, and impacted canine eruption [8].

DNA analysis in forensic identification

Because dental tissues are resistant to environmental conditions like burning, submersion, physical stress, and decomposition, they make a good source of DNA. DNA analysis can provide a vital option for identity confirmation in situations where conventional dental identification techniques are impractical. Particularly in circumstances of mass disasters or cases involving highly damaged bodies, antemortem DNA samples derived from personal items such as toothbrushes, hairbrushes, clothing, blood samples, or biopsies can be compared with DNA retrieved from human remains [23].

Age determination in forensic science

The development of crown and root structures, the stage of tooth eruption, and mixed dentition (primary and permanent teeth) are frequently used in forensic dentistry to estimate age. Furthermore, suppose the person has a thorough dental history and possesses distinctive characteristics like tooth decay, malposition, overlapping, rotations, different fillings or restorations, diastemas (gaps), and dentures or dental implants. In that case, dental identification is more successful [24].

Domestic violence and child abuse in forensic odontology

The World Health Organization acknowledges violence as a serious and growing global public health concern. It divides violence into four different categories: neglect, sexual, psychological, and physical [25]. Dentists should be especially concerned about the orofacial region since it can be affected by all types of violence. Abuse-related injuries can take many different forms, including bruising on the lips, face, and neck; lacerations of the labial and buccal mucosa; cracked anterior teeth; and tears to the frenum. These symptoms should lead to additional research and documentation by dental professionals, as they can be important markers of physical abuse [26].

Implants: a phoenix in modern dentistry

For people who have lost all or part of their teeth, dental implants have emerged as a popular treatment option for regaining both function and appearance [6]. Dental implants are becoming a common oral treatment among urban populations. Forensic odontologists must acknowledge dental implants as a trustworthy form of identification, as more dental specialties adopt implantology. Most implants comprise titanium alloys, renowned for their strength and high melting point (over 1600°C), making them especially resistant to harsh environments like fire or decomposition. Scientific approaches, including DNA analysis, fingerprint comparison, and dental record matching, are the mainstay of non-visual victim identification techniques. Extreme temperatures can cause tooth loss even if teeth are extremely resilient. In these situations, the only physical evidence left for identification may be the features of any dental implants that have been found. Titanium is very useful in forensic identification because of its physical characteristics, which include its high melting point, remarkable corrosion resistance, and strong structural strength, especially in situations involving significant physical trauma [27]. In certain instances, a sufferer may have no natural teeth left in their dentition, entirely or partly, from dental implants. The finding of an implant after a postmortem examination strongly implies that the treating dentist most likely took the radiographic data, which may be accessible in addition to other antemortem paperwork.

Dental implant morphological analysis: implications for forensics

Dental evidence is one of the most commonly used forensic identification techniques, along with fingerprinting and DNA profiling. It has several important benefits, including being reasonably priced and human teeth being among the most resilient body parts because of their hardness and resistance to environmental deterioration. They frequently survive war, terrorist assaults, and natural disasters. Furthermore, previous dental records are usually available, and since each person's dental characteristics, such as restorations, alignment, and wear patterns, are distinct, they are extremely trustworthy for identification [28-30].

Due to the low risks and continually declining costs, restoring fully or partially edentulous jaws has been a routine treatment in recent years. Usually, dental implants are categorized according to their characteristics, design, and attachment methods. Based on their design, implants can be classified as subperiosteal, endosteal, transosteal, or epithelial. Epithelial implants are placed into the oral mucosa, whereas transosteal implants incorporate aspects of endosteal and subperiosteal designs. There are two basic attachment mechanisms: osteointegration, in which the implant fuses with the bone, and periodontal fibers, which are largely theoretical because there are no materials that can promote fiber formation. The physical, chemical, mechanical, and biological characteristics of implant materials can be used to categorize them [31,32].

Radiological evaluation is essential in the preoperative and postoperative phases of dental implantology. It aids in assessing the state of the mouth's soft tissues, the position and condition of the remaining teeth, the underlying bone, and the general cleanliness before surgery. Radiological imaging is utilized after surgery to confirm the implant's location and spot any possible issues. Simple radiography can be used for radiological evaluations, but dental panoramic CT or MRI is usually recommended for a more thorough assessment. Either way, all the criteria listed in Table 1 must be fully specified in the imaging [31]. Placing a radiopaque item of known size on the implant or crown's occlusal surface further aids subsequent implant measurements [33,34].

**Table 1:** Criteria for radiological implant assessment Credit: Serrano-Esteban et al., 2023 [31], under CC license

Type of assessment/observation	Timing	Specific details
Anatomical assessment	Preoperative	Position and size of relevant normal anatomical structures (inferior dental canal, mental foramina, incisive foramen and canal, nasal floor), position and shape of the antral region
Pathological assessment	Preoperative	Preexisting pathologies (both soft and hard tissue)
Dental history	Preoperative	Presence of retained roots or buried teeth
Bone morphometry	Preoperative	Quantitative analysis of alveolar crest/basal bone: shape, width, and height
Bone density analysis	Preoperative	Bone density: amount of cortical bone, density of trabecular bone, and size of trabecular spaces
Radiographic evaluation	Post-implant	Perimplant radiolucency - absent if implant is successful, position of the fixture in the bone, fixture relation to neighboring anatomical structures, implant position, and angles
Clinical evaluation (success)	Post-implant	Vertical bone loss - less than 0.2 mm annually (after the first year) if the implant is successful, healing and integration of the fixture in the bone, the fit of the abutment to the fixture, and crown/prosthesis
Complication monitoring	Post-implant	Development of associated diseases, fractures of the implant/prosthesis
Implant specifics	Post-implant	Implant characteristics

Sahiwal developed a model for implant characterization by dividing the implant into three main parts: apical, midbody, and coronal [7,35,36]. For each section, a set of defining characteristics is described as follows: (1) coronal characteristics include the description of the prosthetic interface (e.g., external or internal hex, Morse taper, or other designs), the flange (whether absent, wider than the implant body, a straight transition from the implant to the flange, a smooth flare from the implant body to the prosthetic interface, or elliptical), and any unique features (e.g., a groove below the flange, fine threads on the entire flange, or a grooved apical part of the flare). (2) For midbody characteristics, the midbody can be either threaded (with V-shaped, square, or reverse buttress, and may have grooves or other distinctive features) or non-threaded. It can be tapered or non-tapered, with potential additional unique features (e.g., stepped design, multiple grooves, diamond-shaped matrix, various coatings, an expanding screw in the middle, or very thin or wide threads). (3) Apical characteristics include the shape (e.g., V-shaped, flat, or curved), the type of holes (round, oval), the presence of an apical chamber, and the number and placement of grooves. Unique characteristics may include two rows of holes, alternating holes and grooves, grooves that extend into the body, dimples, or an expanding screw in the center. For the most common types of implants, refer to Table 2 for a visual guide.

**Table 2:** Main morphological characteristics of dental implants Credit: Serrano-Esteban et al., 2023 [31], under CC license

Shape category	Specific shape/thread type
Body shape	Cylinder
	Tapered
Body thread type	V-shaped
	Reverse buttress
Cervical transition	Wider than the body
	Straight from the body
	Narrow from the body
Cervical neck	Angled
Apex shape	Domed
	Cone
	Flat
	Flared

Types of implant restoration

Cement-Retained Implant Restoration

Despite screw-retained implants showing a lower risk of peri-implant issues, cement-retained options remain more prevalent. This preference is largely due to their superior aesthetics, offering a more natural-looking restoration. Furthermore, dentists often favor cement-retained implants to avoid complications commonly associated with screw-retained systems, such as screw loosening or access hole visibility, contributing to their continued widespread use in dental practice [37].

Implant-Retained Implant Restoration

Issues with screw-retained implants include screw loosening, challenges with implant angulation and achieving a passive fit, and screw access holes. These holes can weaken the overlying restoration, increasing the potential for fractures. Moreover, the intricate components and complex laboratory procedures associated with screw-retained implants often translate to higher costs and a more demanding workflow. In contrast, cement-retained restorations typically offer easier access to posterior regions of the mouth and frequently provide a more seamless and aesthetically pleasing outcome for the patient [38,39].

Role of dental implants in forensic identification in the United States

When it comes to identifying missing or unidentified people, forensic dentistry can significantly benefit from the expertise of dental implantology. This specialist field has frequently been undervalued despite its enormous potential. When used appropriately, dental implantology can significantly increase and broaden the range of forensic identification techniques [40].

A recent case in California demonstrated the vital role of dental implants in forensic identification. A mandible discovered on a beach was found to contain a dental implant. Thanks to cooperation from industry professionals, X-rays helped forensic experts identify the implant’s type, manufacturer, and approximate production year. This process, which can narrow down over 185,000 prosthetic dentists to just a few hundred based on implant brand, highlights the power of implant traceability. If serial numbers were mandatorily etched onto implants, identification could be achieved in minutes, even pinpointing a single device. With the increasing number of implants placed across the U.S., law enforcement and forensic investigators must consider them in identifying unknown remains. Establishing a centralized database, possibly under the FBI or another international body, along with standardized record-keeping protocols, could significantly streamline this process. While the identification numbers aren't visible to the naked eye, they can be read using specialized devices available to medical examiners and manufacturers [40].

Dental implant radiography recognition is a tool for identifying deceased persons. A deceased person's official identification is supported by substantial evidence [41,42]. Forensic odontology is frequently used as the main scientific identification technique in cases with one or more fatalities [43-47]. As dental implants gain popularity as a treatment option, they will probably be more prominent in a person's antemortem records. In certain situations, they may even be the only natural teeth left in a person's dentition [47-49]. Identifying specifics of implant bodies in deceased people can be done in several ways, such as by performing panoramic radiography, taking intraoral X-rays, employing 3D imaging methods like CT scans, and physically removing the implants for visual inspection and direct measurement. There are numerous benefits to intraoral radiography of implant bodies, particularly as odontologists frequently utilize it for postmortem investigations. Intraoral radiographic imaging can be performed using portable radiography equipment in a large-scale catastrophe victim identification scenario where a temporary mortuary is set up [50]. The main disadvantage of identifying implants with intraoral radiography is the distortion and enlargement of images that the projection geometry brings. While the long cone paralleling technique produces less distortion, the bisecting angle technique makes the most noticeable changes in image size [51].

In 2006, Michelinakis et al. developed computer software designed to aid in the identification of dental implants in vivo by allowing users to input various implant characteristics [52]. They compiled a database featuring 87 implant manufacturers, detailing features such as implant shape, surface texture, presence of threads, and variations in the coronal section. These parameters were also included since most dental implant systems come in multiple widths, typically four to five lengths. The Implant Recognition System (IRS) software uses drop-down menus to guide users through categories like implant type, description, threading, surface, collar, diameter, and length. Once the user inputs the known details, the system generates a list of potential matches, including the manufacturer and implant model [52].

To assess the effectiveness of radiographic implant detection and determine whether postmortem assaults and various operator pictures impact the outcomes, a more thorough investigation comparing antemortem and postmortem images of the same implants would be helpful [53]. Recognizing dental implants could help dental operators maintain patients' prosthodontic restorations or in forensic odontology identification casework. Despite having a low success rate, this study demonstrated that certain implants might be recognized from nonstandardized radiographs. Success was mostly ascribed to each examiner's past knowledge. The use of the current IRS system somewhat facilitated recognition.

Utilization of the Implant Recognition Software for dental implant identification

Because of increased patient mobility and the wide variety of implant systems with different designs used worldwide, it might be challenging to identify dental implant systems in patients for whom data are unavailable. The feasibility of employing radiographic imaging techniques to identify implants while still in place has been investigated in several investigations [7,53,54]. More than 220 implant brands made by roughly 80 manufacturers were found after a thorough, critical evaluation of the study data and a systematic search of promotional materials and websites [55]. Identifying the specific dental implant system used in a patient can be a significant challenge for dentists, especially given the wide variety of systems available, each often coming in multiple widths, lengths, and surface coatings. This complexity makes accurate identification even more difficult. When patients lack proper documentation, a considerable amount of clinical time is often spent investigating, relying on the dentist's experience, input from colleagues, or assistance from implant company representatives. This process is not only time-consuming but can also be costly. As dental implantology continues to grow, particularly in implant maintenance, more general dentists, who may not have placed the original implants, will be required to provide ongoing care for these fixtures. Ideally, patients would receive a "passport" at the time of placement detailing the implant system and its specifications. Unfortunately, such documentation is not commonly provided, making it crucial for dentists and dental technicians to accurately identify the type of implant used through other means [29].

Over the past decade, dental implant procedures have evolved significantly, from highly specialized treatments performed by a limited number of dentists to widely accessible services offered by numerous practitioners around the globe. The significant increase in the implant markets around the world is a reflection of this quick expansion. More than 30 implant systems are commercially marketed in the UK alone, and more than 230 different systems are available worldwide. This wide range of choices highlights how difficult it is to diagnose and manage dental implants in various clinical contexts [56-58]. Following the identification of a company that manufactures implants, all pertinent data about its dental implant products were gathered from its webpage (Table 3) [29].

**Table 3:** Variables related to dental implant design and surface properties evaluated in this review Credit: Saghiri et al., 2021 [29], under CC license

Details of the implant data considered in this review
Implant type (implant body shape)
Implant description (abutment connection)
Threaded or not
Surface type
Collar (polished or not)
Diameter
Length

The results are summarized in Tables 4-5, which show the nation where the manufacturer is based, the most common implant body form, surface feature, and abutment connection.

**Table 4:** Number of dental implant manufacturers per country Credit: Saghiri et al., 2021 [29], under CC license

Number of manufacturing companies	Countries
18	USA
13	Germany
10	Italy
7	Spain
5	France
4	Canada, Switzerland, Brazil
3	Israel
2	UK, Japan, Sweden, Finland, Hungary, Korea Republic
1	Czech Republic, South Africa, Bulgaria, Austria, Argentina, Netherlands

**Table 5:** Distribution of implant design characteristics RBM: resorbable blast media, TPS: titanium plasma sprayed, HA: hydroxyapatite Credit: Saghiri et al., 2021 [29], under CC license

Implant body shape	Surface type	Abutment connection
109 cylindrical implants (47.5%)	Etched (23%)	95 external hexagonal connection (41%)
58 bullet implants (25%)	Sandblasted/gritblasted (18%)	66 internal hexagonal connection (28.5%)
52 tapered implants (22.5%)	RBM (6%)	25 Morse taper connection (11%)
7 stepped cylindrical implants (3%)	TPS (9%)	20 not known/other (8.5%)
5 not known/other (2%)	HA coated (17%)	19 one piece (8.5%)
Data not available	Machined (10%)	6 internal octagonal connection (2.5%)
Data not available	Not known/other (17%)	Data not available

The current dental implant software, the Implant Recognition Software, was created using several questions to identify potential implant systems from a dataset maintained in a stand-alone database. Even if not all the information is known, the database will be searched after the nine questions have been answered for implant systems that satisfy the specified requirements [29].

In addition to having potential applications in forensic dentistry, the Implant Recognition Software should make it easier for technicians and doctors to detect any dental implant systems that a patient may present with [29].

Survival of batch numbers within dental implants after incineration

An artificial tooth, crown, or bridge can be securely attached to a dental implant, a prosthetic device that is surgically inserted into the upper or lower jawbone. Titanium, a substance renowned for its strength, resilience, and superior biocompatibility with human bone, is used to make the majority of dental implants [59]. Some manufacturers have recently used zirconia or a titanium-zirconia mix to create dental implants. Zirconia is prized for its corrosion resistance, biocompatibility, and aesthetic qualities, including its tooth-like hue. These recent materials provide alternatives for patients sensitive to metal or who prefer a more natural look [31,60,61]. With more than 460 implant types available to dentists, titanium implantation has expanded globally. The number of implants placed in patients is increasing by more than 1% annually in several nations [62].

**Figure 2:**
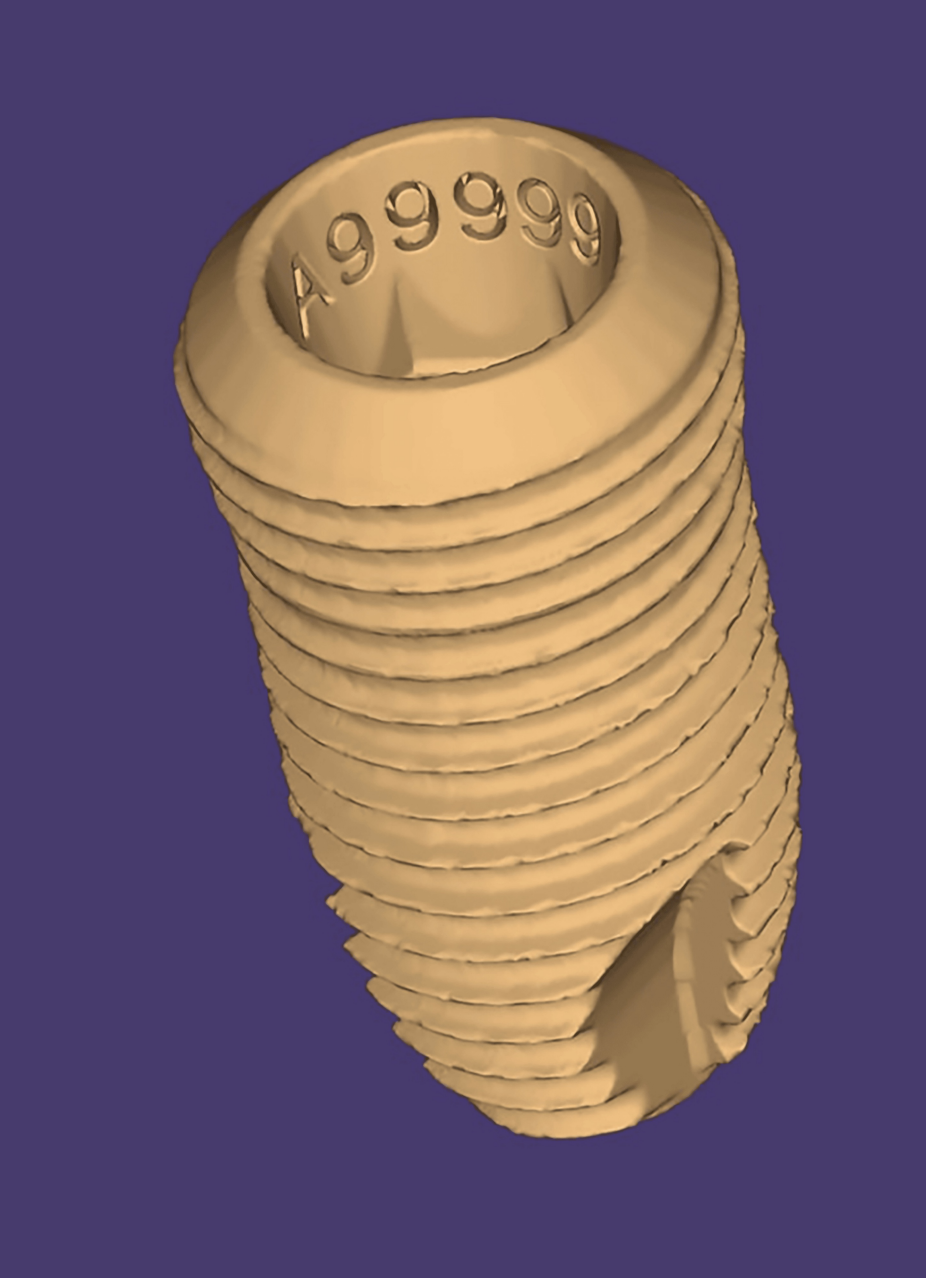
Batch number clearly visible within the implant before incineration Note: This figure has been drawn using the premium version of BioRender (https://BioRender.com/3ilcw8d), agreement license number TA287OFIMS, accessed April 24th, 2025 [6]. Illustration Credit: Dr. Vishnu Desai. Permission granted for the use.

Intense heat obliterates teeth and standard dental materials, hindering victim identification. While implants resist thermal damage due to their physical properties, their mass-produced nature limits their uniqueness. However, incorporating batch numbers (Figure 2) within implants, which this study shows can survive extreme heat, enhances their forensic value. Persuading companies to include unique serial numbers on each implant could revolutionize deceased person identification [61].

Extreme heat can destroy teeth, conventional dental restorative materials, and other commonly used scientific identifiers in victims. However, dental implants are more resistant to thermal damage due to their durable physical properties. Despite this resilience, their use in identification is limited by the lack of uniqueness in mass-produced implants. Introducing batch numbers into implants and ensuring these identifiers remain intact even after exposure to high temperatures would significantly enhance their value as forensic evidence in such situations [61].

Forensic identification using dental implants after incineration

Implants withstand incineration and retain serial numbers etched on their surfaces, which is critical for traceability [61]. Advanced imaging techniques and software-based comparisons enhance identification accuracy, especially when antemortem records are available. While Australia has led significant research in this area, particularly through the University of Adelaide, challenges remain in global standardization, collaboration with manufacturers, and consistent clinical documentation. Integrating traceable elements in implant design could represent a transformative tool in forensic odontology [63].

Future research perspectives

Future research should prioritize building standardized implant databases and advancing implant recognition technologies, leveraging the power of AI. AI can efficiently automate data extraction from diverse records using natural language processing and optical character recognition, populate databases, and minimize errors. AI-driven image recognition (convolutional neural networks) can analyze dental radiographs to identify implant features. Machine learning algorithms can then accurately match these features against the database, improving identification rates. AI can also standardize data, predict identification success, ensure data quality, and create user-friendly search tools, ultimately strengthening forensic capabilities. Research into the durability of various implant materials under extreme conditions and collaboration with manufacturers for implant traceability will further enhance their forensic value.

Limitations

As a narrative review, this article is limited by its reliance on existing literature, which may not comprehensively represent all current practices or technological advancements in implant-based forensic identification. The absence of primary data or case analysis restricts the ability to draw statistically validated conclusions. Additionally, variations in global forensic protocols and documentation standards were not fully explored, which may affect the generalizability of the findings across different regions.

Key features

**Figure 3:**
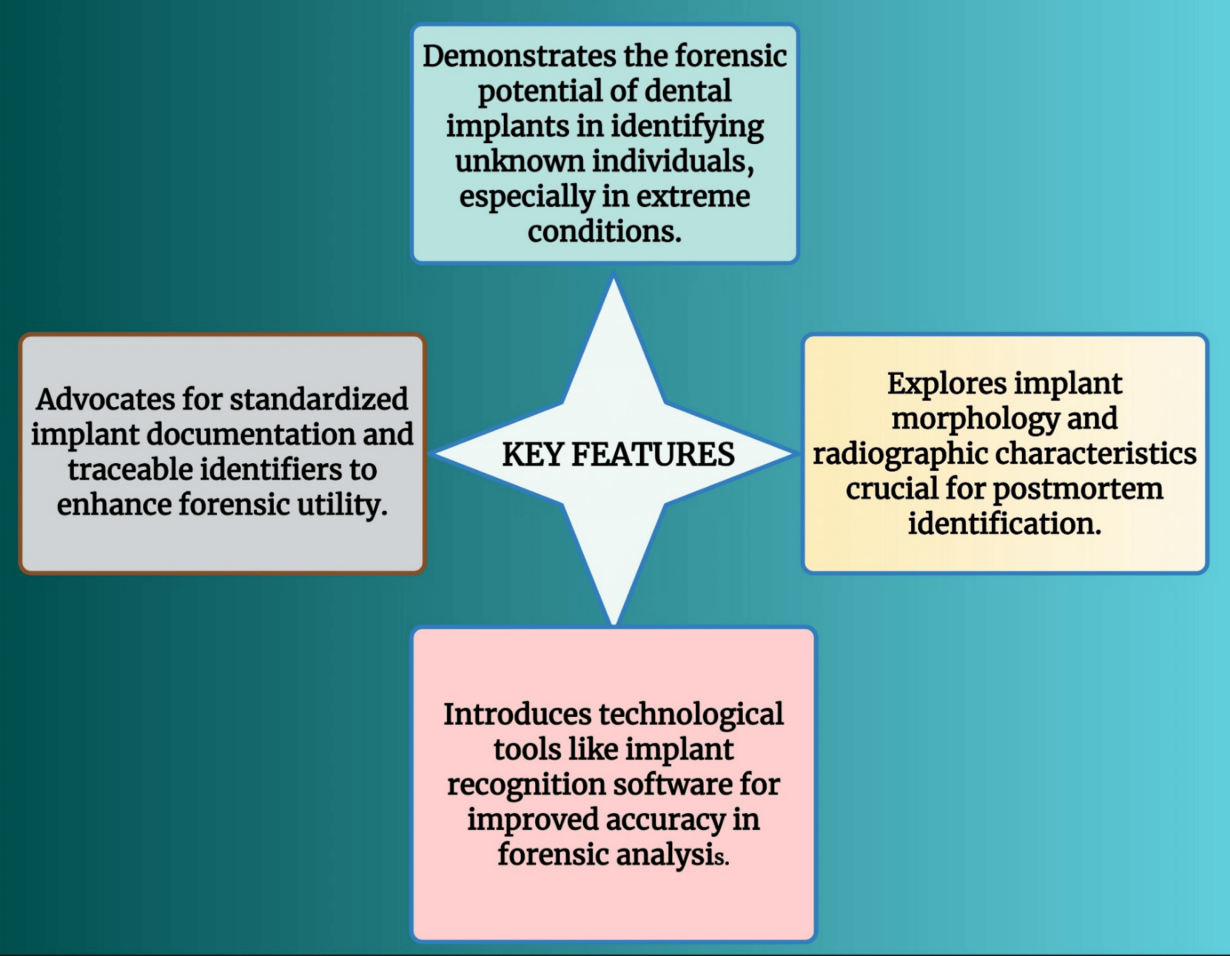
Key features of this narrative review Note: This figure has been drawn using the premium version of BioRender (https://BioRender.com/3ilcw8d), agreement license number JF286R077N, accessed April 24th, 2025 [6]. Illustration Credit: Dr. Vishnu Desai. Permission granted for the use.

This review highlights the vital role of forensic odontology in human identification, focusing on techniques such as bite mark analysis, lip and palatal print studies, radiographic imaging, and DNA profiling. Dental implants, which are appreciated for their longevity, resilience to heat, and traceability via batch or serial numbers, are given special attention. The paper demonstrates the use of Implant Recognition Software for precise identification and presents implant classification based on design criteria. Real-world forensic applications highlight their significance, and the necessity of centralized implant databases and uniform documentation is also emphasized (Figure 3).

## Conclusions

Forensic odontology's use of dental implants marks a substantial breakthrough in recognizing unidentified people, particularly in situations involving mass disasters, fire, or decomposition. Implants are dependable identifiers when other techniques fail because of their distinctive morphological characteristics, long-term stability, and strong resistance to environmental harm. Introducing radiography databases and implant identification software has considerably improved the capacity to link postmortem discoveries with antemortem records. Notwithstanding these developments, there are still issues, such as the requirement for uniform record-keeping, worldwide cooperation between implant makers and forensic specialists, and universal implant documentation. Dental implants have the potential to become a vital tool in forensic science with continued research and advancements in technology, offering precise, effective, and long-lasting solutions for human identification.

## References

[1] Rai B, Kaur J (2013). Evidence-Based Forensic Dentistry.

[2] Chugh A, Narwal A (2017). Oral mark in the application of an individual identification: from ashes to truth. J Forensic Dent Sci.

[3] Toolson LB, Taylor TD (1989). Method for denture identification. J Prosthet Dent.

[4] Kosa F, Antal A, Farkas I (1990). Electron probe microanalysis of human teeth for the determination of individual age. Med Sci Law.

[5] Deepalakshmi TK, Prabhakar M (2014). Role of dental implants in forensic identification. J Forensic Dent Sci.

[6] (2025). BioRender. https://www.biorender.com.

[7] Sahiwal IG, Woody RD, Benson BW, Guillen GE (2002). Radiographic identification of threaded endosseous dental implants. J Prosthet Dent.

[8] Jeddy N, Ravi S, Radhika T (2017). Current trends in forensic odontology. J Forensic Dent Sci.

[9] Pramod JB, Marya A, Sharma V (2012). Role of forensic odontologist in post mortem person identification. Dent Res J (Isfahan).

[10] Debnath N, Gupta R, Nongthombam R, Chandran P (2016). Forensic odontology. J Med Soc.

[11] Mishra SK, Mahajan H, Sakorikar R, Jain A (2014). Role of prosthodontist in forensic odontology. A literature review. J Forensic Dent Sci.

[12] Meinl A, Huber CD, Tangl S, Gruber GM, Teschler-Nicola M, Watzek G (2008). Comparison of the validity of three dental methods for the estimation of age at death. Forensic Sci Int.

[13] Schuller-Götzburg P, Suchanek J (2007). Forensic odontologists successfully identify tsunami victims in Phuket, Thailand. Forensic Sci Int.

[14] Shamim T, Varghese VI, Shameena P, Sudha S (2006). Human bite marks: the tool marks of the oral cavity. J Indian Acad Forensic Med.

[15] Vilborn P, Bernitz H (2022). A systematic review of 3D scanners and computer assisted analyzes of bite marks: searching for improved analysis methods during the Covid-19 pandemic. Int J Legal Med.

[16] Martin-de las Heras S, Valenzuela A, Ogayar C, Valverde AJ, Torres JC (2005). Computer-based production of comparison overlays from 3D-scanned dental casts for bite mark analysis. J Forensic Sci.

[17] Rao DS, Ali IM, Annigeri RG (2016). Bitemarks - A review. J Dent Res Rev.

[18] Balachander N, Babu NA, Jimson S, Priyadharsini C, Masthan KM (2015). Evolution of forensic odontology: an overview. J Pharm Bioallied Sci.

[19] Venkatesh R, David MP (2011). Cheiloscopy: an aid for personal identification. J Forensic Dent Sci.

[20] Reddy LV (2011). Lip prints: an overview in forensic dentistry. J Adv Oral Res.

[21] Nagare SP, Chaudhari RS, Birangane RS, Parkarwar PC (2018). Sex determination in forensic identification, a review. J Forensic Dent Sci.

[22] Sha SK, Rao BV, Rao MS, Kumari KV, Chinna SK, Sahu D (2017). Are tooth prints a hard tissue equivalence of finger print in mass disaster: a rationalized review. J Pharm Bioallied Sci.

[23] Pawar RK, More CB (2018). Sex determination from tooth pulp deoxyribonucleic acid using polymerase chain reaction. J Forensic Dent Sci.

[24] Asami R, Aboshi H, Iwawaki A, Ohtaka Y, Odaka K, Abe S, Saka H (2019). Age estimation based on the volume change in the maxillary premolar crown using micro CT. Leg Med (Tokyo).

[25] (2002). World report on violence and health. https://www.who.int/publications/i/item/9241545615.

[26] Eggensperger N, Smolka K, Scheidegger B, Zimmermann H, Iizuka T (2007). A 3-year survey of assault-related maxillofacial fractures in central Switzerland. J Craniomaxillofac Surg.

[27] Dhakshaini M, Satpathy A (2014). Contribution of a prosthodontist in the field of forensic odontology. Int J Prosthodont Restor Dent.

[28] Bóscolo FN, Almeida SM, Haiter Neto F, Oliveira AEF, Tuji FM (2002). Fraudulent use of radiographic images. J Forensic Odontostomatol.

[29] Saghiri MA, Freag P, Fakhrzadeh A, Saghiri AM, Eid J (2021). Current technology for identifying dental implants: a narrative review. Bull Natl Res Cent.

[30] Piciorus I, Capatina C, Sirbu A, Hostiuc S (2008). Accessory bones and growth cartilages: sources of errors in forensic trauma assessment. Romanian J Leg Med.

[31] Serrano-Esteban AI, Requena-Gómez E, Mena-Alvarez J, Rodríguez C, Bufalá-Pérez M, Aragoneses JM (2023). Cadaveric identification through macroscopic analysis of dental implants subjected to high temperatures-an experimental model. J Funct Biomater.

[32] Sanchez P (2003). Phillips' Science of Dental Materials - Phillip Anusavice. https://www.academia.edu/41764796/Phillips_Science_of_Dental_Materials_Phillip_Anusavice.

[33] Hostiuc S, Curcă GC, Dermengiu D, Rusu MC (2008). Bitemark analysis in legal medicine - literature review. Romanian J Leg Med.

[34] Berketa JW, Hirsch RS, Higgins D, James H (2010). Radiographic recognition of dental implants as an aid to identifying the deceased. J Forensic Sci.

[35] Rai B, Cameriere R, Ferrante L (2009). Accuracy of Cameriere et al regression equation in Haryana population. Romanian J Leg Med.

[36] Sahiwal IG, Woody RD, Benson BW, Guillen GE (2002). Macro design morphology of endosseous dental implants. J Prosthet Dent.

[37] Wadhwani C, Hess T, Faber T, Piñeyro A, Chen CSK (2010). A descriptive study of the radiographic density of implant restorative cements. J Prosthet Dent.

[38] Hebel KS, Gajjar RC (1997). Cement-retained versus screw-retained implant restorations: achieving optimal occlusion and esthetics in implant dentistry. J Prosthet Dent.

[39] Jain JK, Sethuraman R, Chauhan S, Javiya P, Srivastava S, Patel R, Bhalani B (2018). Retention failures in cement- and screw-retained fixed restorations on dental implants in partially edentulous arches: a systematic review with meta-analysis. J Indian Prosthodont Soc.

[40] Takahashi T, Nozaki K, Gonda T, Mameno T, Wada M, Ikebe K (2020). Identification of dental implants using deep learning-pilot study. Int J Implant Dent.

[41] Rothwell BR (2001). Principles of dental identification. Dent Clin North Am.

[42] Hinchliffe J (2011). Forensic odontology, part 1. Dental identification. Br Dent J.

[43] James H (2005). Thai tsunami victim identification - overview to date. J Forensic Odontostomatol.

[44] Valenzuela A, Martin-de las Heras S, Marques T, Exposito N, Bohoyo JM (2000). The application of dental methods of identification to human burn victims in a mass disaster. Int J Legal Med.

[45] Hutt JM, Ludes B, Kaess B, Tracqui A, Mangin P (1995). Odontological identification of the victims of flight AI. IT 5148 air disaster Lyon-Strasbourg 20.01.1992. Int J Legal Med.

[46] Solheim T, Lorentsen M, Sundnes PK, Bang G, Bremnes L (1992). The "Scandinavian Star" ferry disaster 1990--a challenge to forensic odontology. Int J Legal Med.

[47] Andersen L, Juhl M, Solheim T, Borrman H (1995). Odontological identification of fire victims--potentialities and limitations. Int J Legal Med.

[48] Simpson EK, James RA, Eitzen DA, Byard RW (2007). Role of orthopedic implants and bone morphology in the identification of human remains. J Forensic Sci.

[49] Nuzzolese E, Lusito S, Solarino B, Di Vella G (2008). Radiographic dental implants recognition for geographic evaluation in human identification. J Forensic Odontostomatol.

[50] Hermsen KP, Jaeger SS, Jaeger MA (2008). Radiation safety for the NOMAD portable X-ray system in a temporary morgue setting. J Forensic Sci.

[51] Biggerstaff RH, Phillips JR (1976). A quantitative comparison of paralleling long-cone and bisection-of-angle periapical radiography. Oral Surg Oral Med Oral Pathol.

[52] Michelinakis G, Sharrock A, Barclay CW (2006). Identification of dental implants through the use of Implant Recognition Software (IRS). Int Dent J.

[53] Korkchi M, Lekholm U, Dahlbom U, Borrman H (1995). Accuracy in identification of implant treated patients by use of intraoral radiographs. J Forensic Odontostomatol.

[54] Sahiwal IG, Woody RD, Benson BW, Guillen GE (2002). Radiographic identification of nonthreaded endosseous dental implants. J Prosthet Dent.

[55] (2003). European markets for dental implants. Implant Dent.

[56] Jokstad A, Braegger U, Brunski JB, Carr AB, Naert I, Wennerberg A (2003). Quality of dental implants. Int Dent J.

[57] (2003). Japanese markets for dental implants. Implant Dent.

[58] (2001). U.S. markets for dental implants 2001: executive summary. Implant Dent.

[59] Bhuvaneswaran M (2010). Principles of smile design. J Conserv Dent.

[60] Sennerby L, Dasmah A, Larsson B, Iverhed M (2005). Bone tissue responses to surface-modified zirconia implants: a histomorphometric and removal torque study in the rabbit. Clin Implant Dent Relat Res.

[61] Berketa J, James H, Marino V (2010). Survival of batch numbers within dental implants following incineration as an aid to identification. J Forensic Odontostomatol.

[62] Elani HW, Starr JR, Da Silva JD, Gallucci GO (2018). Trends in dental implant use in the U.S., 1999-2016, and projections to 2026. J Dent Res.

[63] Gambini L, Fonseca GM (2022). Dental implants for forensic identification in cremations: recommendations from a systematic review (Article in Spanish). Odontoestomatol.

